# Supplementary material for: MetaRibo-Seq measures translation in microbiomes
Source: Nat Commun. 2020 Jun 29;11:3268. doi: 10.1038/s41467-020-17081-z (PMC7324362; doi:10.1038/s41467-020-17081-z)
Supplement: Supplementary file 10 — Supplementary Data 7 [file 41467_2020_17081_MOESM10_ESM.zip › File2/Confidence_VeryHigh_Taxonomy/223010_out.krona.html]

Javascript must be enabled to view this page.

members
magnitude
magnitudeUnassigned
count
unassigned
taxon
rank

223010\_out

14

superkingdom
2
14

phylum
1239
14

class
186801
14

order
186802
14

1950913
species
6

SRS013965\_contig\_number\_26237SRS075984\_contig\_number\_contig-100\_7378.46913SRS077194\_contig\_number\_contig-100\_5091.43897SRS077392\_contig\_number\_contig-100\_14329.171304SRS1041132\_contig\_number\_contig-100\_6721.6722SRS144506\_contig\_number\_contig-100\_28722.136027

8

SRS015431\_contig\_number\_contig-100\_5923.5924SRS019068\_contig\_number\_59819SRS020328\_contig\_number\_contig-100\_3312.179350SRS042966\_contig\_number\_contig-100\_25391.66460SRS063985\_contig\_number\_contig-100\_21832.21832SRS064276\_contig\_number\_18373SRS104400\_contig\_number\_58026SRS148159\_contig\_number\_29827
1898207
species
